# Supplementary material for: Sorting at embryonic boundaries requires high heterotypic interfacial tension
Source: Nat Commun. 2017 Jul 31;8:157. doi: 10.1038/s41467-017-00146-x (PMC5537356; doi:10.1038/s41467-017-00146-x)
Supplement: Supplementary file 2 — Supplementary Software 1 [file 41467_2017_146_MOESM2_ESM.zip › PottsModel/SrcPottsModel/doc/model/ProtrusiveCell.html]

ProtrusiveCell


---


|  |  |  |
| --- | --- | --- |
| **Overview** \| **Package** \| **Class** \| **Use** \| **Tree** \| **Deprecated** \| **Index** \| **Help** | |  |
| **PREV CLASS**   **NEXT CLASS** | **FRAMES**    **NO FRAMES**     **All Classes** |  |
| SUMMARY: NESTED \| FIELD \| CONSTR \| METHOD | DETAIL: FIELD \| CONSTR \| METHOD |  |


---


## model Class ProtrusiveCell

```
java.lang.Object
  mvc.AObservable
      model.Cell
          model.ProtrusiveCell
```

**All Implemented Interfaces:**: KnowsConstants, IObservable

---

``` public class ProtrusiveCell extends Cell ```

---

**Nested Class Summary**

**Nested classes/interfaces inherited from class model.Cell**

`Cell.CellType, Cell.Energy, Cell.State`


| **Field Summary** | |
| --- | --- |
| `private  CellDisplay` | `aCellDisplay` |
| `private  java.awt.Color[]` | `aColorScale` |
| `private  ProtrusivePixel` | `aTargetBoundaryPixel` |
| `private  HalfLine` | `aTargetVector` |
| `private  int` | `cColorBins` |
| `private  float` | `rotateRatio` |

**Fields inherited from class model.Cell**

`aConstants, cAreaUnit, cEdgeUnit, cNotificationSource`


**Constructor Summary**

`ProtrusiveCell(Cell.CellType pType,
int pID,
Lattice pLattice)`


| **Method Summary** | |
| --- | --- |
| `Arrow` | `getArrow()` |
| `java.awt.Color` | `getColorFromScale(double index)` |
| `CellDisplay` | `getDisplay()` |
| `ProtrusivePixel` | `getTargetBoundaryPixel()` |
| `Vector` | `getTargetVector()`             Returns vector between current center of mass |
| `void` | `initalize()` |
| `void` | `updateParameters(Pixel pPixel, boolean pIsAdded)`             Call after a pixel has been added or removed from a cell |

**Methods inherited from class model.Cell**

`addPixel, adjustCom, adjustedArea, adjustedComX, adjustedComY, adjustedPerimeter, equals, getArea, getAreaEnergy, getAreaEnergy, getCellTypeColor, getCellTypeColor, getCellTypeColorName, getCellTypeFromID, getCenterOfMass, getComX, getComY, getConstants, getID, getInformation, getInteractionEnergy, getLocalAreaEnergy, getLocalPerimeterEnergy, getNotificationSource, getPerimeter, getPerimeterEnergy, getPerimeterEnergy, getPixel, getPixels, getRandomBoundaryPixel, getRandomPixel, getShapeMetric, getType, hashCode, isInitialized, isMedium, removePixel, toString`

**Methods inherited from class mvc.AObservable**

`addObserver, getNotificationSequenceNumber, notifyObservers, removeAllObservers, removeObserver`

**Methods inherited from class java.lang.Object**

`clone, equals, finalize, getClass, notify, notifyAll, wait, wait, wait`

**Field Detail**

### aTargetVector

```
private final HalfLine aTargetVector
```

---


### aTargetBoundaryPixel

```
private ProtrusivePixel aTargetBoundaryPixel
```

---


### aCellDisplay

```
private final CellDisplay aCellDisplay
```

---


### aColorScale

```
private final java.awt.Color[] aColorScale
```

---


### cColorBins

```
private final int cColorBins
```

**See Also:**: Constant Field Values

---


### rotateRatio

```
private final float rotateRatio
```

**See Also:**: Constant Field Values


**Constructor Detail**

### ProtrusiveCell

```
public ProtrusiveCell(Cell.CellType pType,
                      int pID,
                      Lattice pLattice)
```


**Method Detail**

### getColorFromScale

```
public java.awt.Color getColorFromScale(double index)
```

---


### initalize

```
public void initalize()
```

:   **Overrides:**: `initalize` in class `Cell`

---


### getDisplay

```
public CellDisplay getDisplay()
```

:   **Overrides:**: `getDisplay` in class `Cell`

---


### getArrow

```
public Arrow getArrow()
```

---


### getTargetVector

```
public Vector getTargetVector()
```

:   Returns vector between current center of mass

    :   **Returns:**

---


### updateParameters

```
public void updateParameters(Pixel pPixel,
                             boolean pIsAdded)
```

:   **Description copied from class: `Cell`**
:   Call after a pixel has been added or removed from a cell

    :   **Overrides:**: `updateParameters` in class `Cell`

---


### getTargetBoundaryPixel

```
public ProtrusivePixel getTargetBoundaryPixel()
```


---


|  |  |  |
| --- | --- | --- |
| **Overview** \| **Package** \| **Class** \| **Use** \| **Tree** \| **Deprecated** \| **Index** \| **Help** | |  |
| **PREV CLASS**   **NEXT CLASS** | **FRAMES**    **NO FRAMES**     **All Classes** |  |
| SUMMARY: NESTED \| FIELD \| CONSTR \| METHOD | DETAIL: FIELD \| CONSTR \| METHOD |  |


---
